# Structural characterization of a ribose-5-phosphate isomerase B from the pathogenic fungus *Coccidioides immitis*

**DOI:** 10.1186/1472-6807-11-39

**Published:** 2011-10-13

**Authors:** Thomas E Edwards, Ariel B Abramov, Eric R Smith, Ruth O Baydo, Jess T Leonard, David J Leibly, Kaitlin B Thompkins, Matthew C Clifton, Anna S Gardberg, Bart L Staker, Wesley C Van Voorhis, Peter J Myler, Lance J Stewart

**Affiliations:** 1 Seattle Structural Genomics Center for Infectious Disease (SSGCID), USA; 2 Emerald BioStructures, 7869 NE Day Road West, Bainbridge Island, WA, 98110, USA; 3 Seattle Biomedical Research Institute, 307 Westlake Avenue North, Suite 500, Seattle, WA 98109, USA; 4 School of Medicine, University of Washington, 1959 N.E. Pacific Avenue, MS 356423, Seattle, WA 98195-7185, USA; 5 Departments of Global Health and Medical Education & Biomedical Informatics, University of Washington, Box 357230, Seattle, WA 98195, USA

## Abstract

**Background:**

Ribose-5-phosphate isomerase is an enzyme that catalyzes the interconversion of ribose-5-phosphate and ribulose-5-phosphate. This family of enzymes naturally occurs in two distinct classes, RpiA and RpiB, which play an important role in the pentose phosphate pathway and nucleotide and co-factor biogenesis.

**Results:**

Although RpiB occurs predominantly in bacteria, here we report crystal structures of a putative RpiB from the pathogenic fungus *Coccidioides immitis*. A 1.9 Å resolution apo structure was solved by combined molecular replacement and single wavelength anomalous dispersion (SAD) phasing using a crystal soaked briefly in a solution containing a high concentration of iodide ions. RpiB from *C. immitis *contains modest sequence and high structural homology to other known RpiB structures. A 1.8 Å resolution phosphate-bound structure demonstrates phosphate recognition and charge stabilization by a single positively charged residue whereas other members of this family use up to five positively charged residues to contact the phosphate of ribose-5-phosphate. A 1.7 Å resolution structure was obtained in which the catalytic base of *C. immitis *RpiB, Cys76, appears to form a weakly covalent bond with the central carbon of malonic acid with a bond distance of 2.2 Å. This interaction may mimic that formed by the suicide inhibitor iodoacetic acid with RpiB.

**Conclusion:**

The *C. immitis *RpiB contains the same fold and similar features as other members of this class of enzymes such as a highly reactive active site cysteine residue, but utilizes a divergent phosphate recognition strategy and may recognize a different substrate altogether.

## Background

Ribose-5-phosphate isomerases catalyze the interconversion of ribulose-5-phosphate and ribose-5-phosphate as an important part of the pentose phosphate pathway [1]. Ribose-5-phosphate is used in nucleotide and co-factor biosynthesis. As with other isomerases, ribose-5-phosphate catalyzes this reaction at near equilibrium. Two types of ribose-5-phosphate isomerases exist, RpiA and RpiB, which share little structural homology and have distinct active sites and mechanisms of action [2]. Most organisms such as *Escherichia coli *contain both RpiA and RpiB, but other organisms contain only one class of Rpi. In *E. coli*, a double *rpia/rpib *knockout exhibited severely impaired growth [3]. RpiB occurs almost exclusively in bacteria.

RpiB is a member of the LacAB_rpiB superfamily of proteins (PFAM PF02502). RpiB from different organisms may have different substrate specificities and several annotated RpiB enzymes act upon allose-5-phosphate rather than or in addition to ribose-5-phosphate and are thus named AlsI. Indeed, one of the first crystal structures solved for a member of this family was the *E. coli *RpiB/AlsI [2]. Given the important cellular role of ribose-5-phosphate isomerases and that mammals only have RpiA, interest has been generated in RpiB enzymes as potential drug targets, especially in pathogenic organisms, many of which only have RpiB. *E. coli *RpiB inhibitors have been described [4] and inhibitor-bound crystal structures have been determined for RpiB from *Mycobacterium tuberculosis *[5] which causes tuberculosis and *Trypanosma cruzi *[6], the causative agent of trypanosomiasis.

*Coccidioides immitis *is a pathogenic fungus that causes coccidioidomycosis, also known as Valley Fever [7-9]. One gene (CIMG_07932) encodes a putative uncharacterized protein that contains high sequence homology with RpiB enzymes from closely related organisms. Specifically, the CIMG_07932 gene product contains 98% sequence identity to an Rpi from *C. posadasii*, 89% sequence identity to an Rpi from the non-pathogenic fungus *Uncinocarpus reesii*, and 78% sequence identity to an RpiB from *Paracoccidioides brasiliensis*. We have undertaken structural analysis of RpiB from *C. immitis *and present here crystal structures in apo and ligand bound forms.

## Results and Discussion

### Structure determination of *C. immitis *RpiB

**Figure 1:**
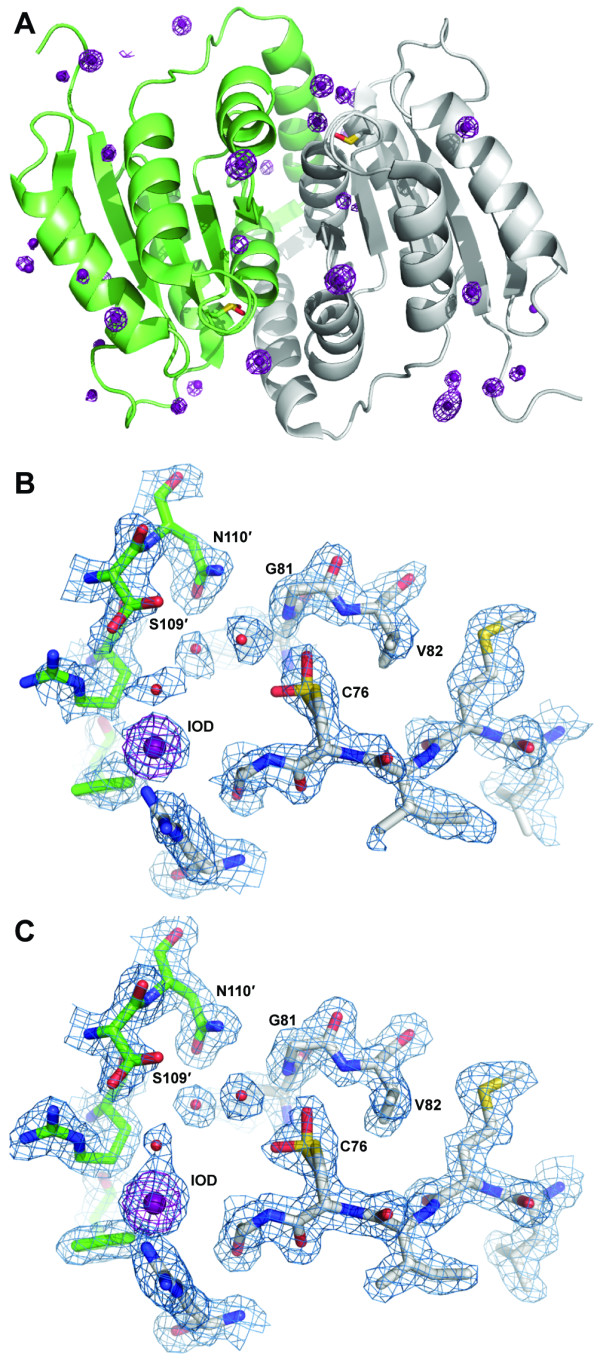
****A **Global fold of *C. immitis *RpiB showing iodide ions (violet spheres) and anomalous difference Fourier map shown in violet mesh contoured at 5.0 σ**. Protomer A is shown in gray ribbons and protomer B is shown in light green ribbons. The oxidized cysteine residues are shown in sticks representation. **B **Experimental electron density map from combined SAD/MR is shown contoured at 1.0 σ, and the anomalous difference Fourier map is shown in violet mesh contoured at 5.0 σ. **C **The active site oxidized cysteine is modeled as Cys-OH (sulfenic acid) in two conformations; the shape of the electron density was not consistent with sulfinic acid or sulfonic acid, the latter of which would have produced major steric clash with the main chain of Gly79 and the side chain of Val82. The 2|F_o_|-|F_c_| map is shown in blue mesh contoured at 1.0 σ.

Given that the *C. immitis *RpiB contains modest sequence identity to other structurally characterized RpiBs, we attempted to solve the crystal structure of *C. immitis *RpiB by iodide ion single wavelength anomalous dispersion (SAD) phasing [10,11], a strategy that has proven successful for structure determination of many SSGCID targets [12,13]. Attempts at phasing entirely with SAD resulted in poor quality experimental phases (FOM of 0.29 prior to density modification). Attempts to solve the structure by molecular replacement with other RpiBs such as that from *Clostridium thermocellum *[14] yielded clear rotation and translation solutions, yet poor refinement statistics (R of 0.39 and R_free _0.47). Addition of the MR solution to a SAD experiment has been shown in improve phase quality [15]. Therefore the partial molecular replacement solution was combined with the SAD phases and yielded a clearly interpretable electron density map (FOM 0.53 prior to density modification) into which Buccaneer [16] built both molecules in the asymmetric unit end-to-end in less than one minute (Table 1; Figure 1).

**Table 1:** Data collection, phasing and refinement statistics

	Iodide	Phosphate	Malonic acid
*Data reduction*			
Space group	*C2*	*F*222	*F*222
Unit-cell parameters	*a *= 103.2 Å, *b *= 49.9 Å, *c *= 62.0 Å, β = 108.6°	*a *= 77.7 Å, *b *= 85.2 Å, *c *= 96.3 Å	*a *= 77.5 Å, *b *= 84.4 Å, *c *= 96.2 Å
Resolution range (Å)	50-1.9 (1.95-1.90)	50-1.8 (1.85-1.80)	50-1.7 (1.74-1.70)
Unique reflections	23,513 (1520)	14,967 (1088)	16,561 (1028)
R_merge_	0.043 (0.286)	0.123 (0.397)	0.044 (0.256)
Mean *I*/σ(*I*)	13.9 (2.9)	17.5 (3.0)	34.7 (6.6)
Completeness	98.9% (87.5%)	99.8% (98.7%)	94.5% (80.2%)
Multiplicity	5.8 (2.9)	6.9 (3.9)	9.6 (5.3)
			
*Phasing*			
Anomalous Correlation	58% (8%)		
SigAno	1.53 (0.81)		
Iodide Sites	21		
FOM (Phaser EP)	0.53		
			
*Refinement*			
R_cryst_	0.166 (0.185)	0.150 (0.212)	0.144 (0.156)
R_free_	0.205 (0.242)	0.176 (0.270)	0.175 (0.189)
RMSD bonds (Å)	0.015	0.015	0.012
RMSD angles (°)	1.364	1.288	1.323
Protein Atoms	2415	1198	1210
Waters	213	173	169
Iodide Ions	29	0	0
Mean *B*-factor (Å^2^)	18.3	13.2	13.7
Reflections	22,275 (1434)	14,170 (1031)	15,677 (968)
R_free_ Reflections	1205	752	832
			
*Validation*			
Ramachandran favored	100%	100%	98.7%
Ramachandran outliers	0%	0%	0%
Molprobity score [35]	1.42 (97^th ^percentile)	0.98 (100^th^)	1.32 (97^th^)
PDB ID	3QD5	3SDW	3SGW

R_free _= Σ_h_||F_obs_| - |F_calc_||/Σ_h_|F_obs_|. Values in parenthesis indicate the values for the highest of twenty resolution shells

R_free _was calculated using 5% of the reflections omitted from the refinement [31].

Interestingly, the active site contains electron density consistent with an oxidized cysteine residue (Cys76) in both protomers (Figure 1). Although the exact oxidation state of the cysteine residue was not determined, the electron density maps were most consistent with two conformations of the sulfenic state rather than sulfinic or sulfonic acid. The oxygen on Cys76 appears to adopt two different orientations contacting the backbone nitrogen and side chain hydroxyl of Thr78 in one orientation and the backbone nitrogens of Gly81 and Val82 in the other. The oxidized nature of Cys76 may imply that this residue, thought to be the catalytic base, is highly reactive. The high concentration solution of sodium iodide used for phasing likely contains iodine, indicated by a light yellow color. Iodine is the most probable source for the oxidation of Cys76, which was not oxidized in the other two *C. immitis *RpiB structures (see below). Oxidation is unlikely to have occurred as a result of radiation damage since the data were collected in house under cryogenic temperatures. Oxidized cysteines have been observed previously in the presence of iodide ions [17,18] and also for other RpiB enzymes (PDB entries 1O1X [19] and 3C5Y, no primary citation) determined in the absence of iodide.

### Comparison with other ribose-5-phosphate isomerases

**Figure 2:**
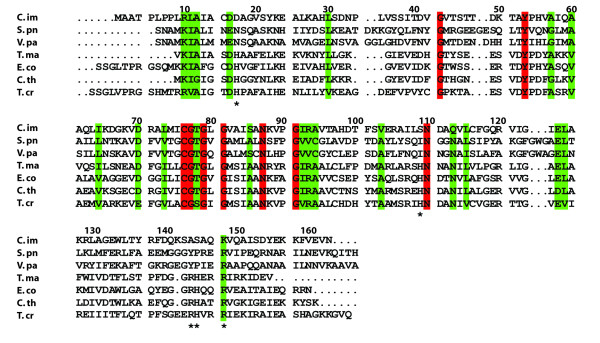
**Multiple sequence alignment of RpiB crystal structures from different organisms. *C. immitis *is from the current study (PDB ID **3QD5). S. pu is *Streptococcus pneumonia *(2PPW, no primary citation), V. pa is *Vibrio parahaemolyticus *(3ONO, no primary citation), T. ma is *Thermotoga maritima *(PDB ID 1O1X) [19], E. co is *E. coli *(1NN4) [2], C. th is *Clostridium thermocellum *(3HEE) [14], T. cr is *Trypanosoma cruzi *(3K8C) [6]. *Mycobacterium tuberculosis *contains different active site residues and utilizes a variant catalytic mechanism [5,22], and thus the sequence is not shown in the alignment. Residues marked with an * are involved in recognition of the phosphate moiety of ribose-5-phosphate. Numbering is based on the *C. immitis *sequence.

The *C. immitis *RpiB contains ~20-38% sequence identity with other structurally characterized RpiB enzymes, although fewer than 10 amino acids are completely conserved across these sequences (Figure 2). The overall structure of the *C. immitis *putative RpiB is quite similar to other structurally characterized RpiB enzymes from bacterial as well as eukaryotic organisms (Table 2). In addition to the conserved overall fold, certain other RpiB features are present in the *C. immitis *RpiB structure such as the highly conserved cis-peptide at residue Gly43.

**Table 2:** Comparison of the global structure of *C. immitis *RpiB with RpiB enzymes from other organisms

Organism	Reference PDB	Similar Cα atoms	R.m.s.d. (Å)	Identity (%)
Bacteria				
*Clostridium thermocellum*	3HEE [14]	148	*1.17*	*34*
*Escherichia coli*	1NN4 [2]	143	*1.10*	*38*
*Mycobacterium tuberculosis*	2VVO [5,22]	141	*1.18*	*26*
*Novosphingobium aromaticivorans*	3C5Y	149	*1.97*	*<20*
*Streptococcus pneumoniae*	2PPW	146	*1.91*	*<20*
*Thermotoga maratima*	1O1X [19]	143	*1.24*	*33*
*Vibrio parahaemolyticus*	3ONO	149	*1.86*	*<20*
				
Eukaryotes				
*Giardia lamblia*	3S5P	125	*1.06*	*28*
*Trypanosoma cruzi*	3K8C [6]	148	*1.40*	*29*

Superposition calculations were done in CCP4 using the program Superposition and the secondary structure matching setting. The number of similar Cα atoms (residues) is reported. For PDB entries 3C5Y (JCSG), 2PPW (Wu, R. et al.), 3ONO (Kim, Y. et al.) and 3S5P (Edwards, T.E. et al.) no primary citation has been reported.

The *C. immitis *RpiB contains the canonical open (α,β) Rossman fold, a common fold for proteins that bind nucleotides or nucleotide-like molecules [20]. As determined by the Protein Interfaces, Surfaces and Assemblies (PISA) server [21], the quaternary structure appears to be dimeric (Figure 1) with 7408 Å^2 ^of buried surface (12,690 Å^2 ^of surface area for the dimer). However, based on the structure, PISA also predicts a tetrameric structure (generated by crystal symmetry) in which 17,610 Å^2 ^of surface area is buried (22,730 Å^2 ^of surface area for the tetramer). The tetrameric structure of *C. immitis *RpiB is most likely a dimer of dimers with one strong dimer and the secondary weaker dimer interaction that makes the tetramer. This quaternary structure is consistent with the two ligand bound *C. immitis *RpiB described below in a different crystal form, and with previous X-ray structural characterization of RpiB, such as that from *E. coli *[2]. Dynamic light scattering (DLS) experiments indicated a monodisperse protein with an approximate molecular weight of 80 kDa which is consistent with a tetramer in solution. DLS measurement on *E. coli *RpiB also indicated a tetramer in solution [2]. Thermofluor analysis indicated a stable protein with a melting temperature of 52°C. The melting temperature was unaffected by the presence of ribose-5-phosphate, ribose-5-phosphate with MnCl_2_, or iodoacetate.

### *C. immitis *RpiB substrate recognition

**Figure 3:**
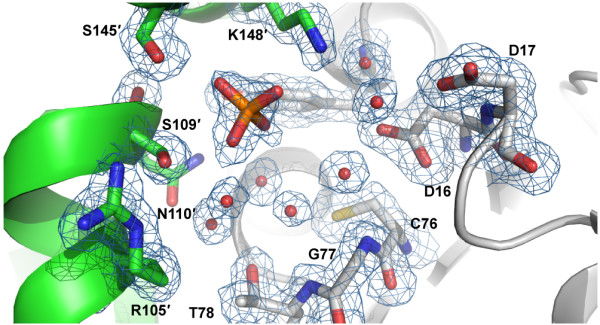
**1.8 Å resolution crystal structure of *C. immitis *RpiB bound to phosphate**. The coloring is the same as used in Figure 1 with one protomer in gray and the other in green. The 2|F_o_|-|F_c_| map is shown in blue mesh contoured at 1.0 σ.

**Figure 4:**
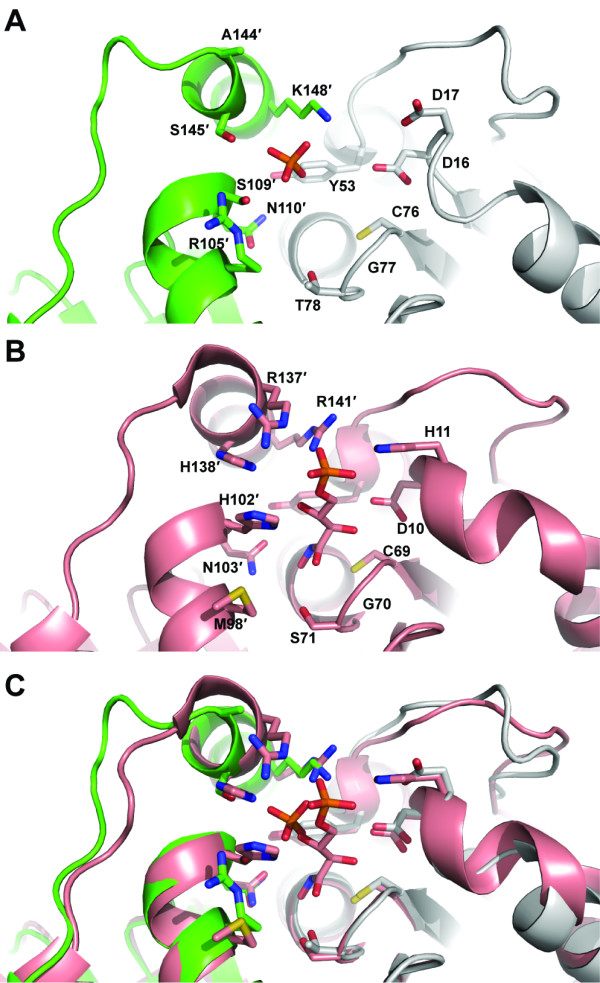
**Comparison of active site residues of eukaryotic RpiB enzymes from *C. immitis *(green and gray in panel A) and *T. cruzi ***[6]**(salmon in panel B) and overlay in panel C**. The *C. immitis *structure contains phosphate whereas the *T. cruzi *structure contains the competitive inhibitor 4-deoxy-4-phospho-D-erythronohydroxamic acid.

Structures of RpiB from other organisms have been obtained in the presence of ribose-5-phosphate or inhibitors. Comparison of the active site in multiple sequence alignments showed that several residues involved in recognition of the phosphate moiety of ribose-5-phosphate or ribulose-5-phosphate are not conserved in *C. immitis *RpiB (Figure 2). While other organisms use up to five positively charged residues to recognized the phosphate moiety, only one of these residues is positively charged (Lys148) in *C. immitis*. Several other residues are small polar residues such as Ser109 and Ser145. Residue 17 is typically a histidine or asparagine, but is surprisingly a negatively charged aspartic acid residue in *C. immitis*. Because of the decreased size of several residues and the presence of Asp17, we speculated that the *C. immitis *RpiB may utilize a cation to facilitate recognition of ribose-5-phosphate. RpiB enzymes from other organisms typically recognize ribose-5-phosphate with a low affinity of K_m _~ 1-5 mM [2,22]. Therefore, we performed co-crystallization experiments in the presence of 20 mM ribose-5-phosphate or 20 mM ribose-5-phosphate with 12 mM MnCl_2_, which are in excess of the protein (~3.3 mM). We obtained a 1.8 Å resolution data set (Table 1) from *C. immitis *RpiB co-crystallized in the presence of 20 mM ribose-5-phosphate and 12 mM MnCl_2_. Despite the presence of 20 mM substrate, we only observed substantial electron density for phosphate bound in the active site, which presumably came from the 0.1 M SPG buffer (succinic acid, phosphate, glycine) buffer at pH 5.0 (Figure 3). Therefore, it appears that under the conditions of the crystallization experiment, phosphate outcompeted ribose-5-phosphate for binding to the active site. Addition electron density extends from one of the oxygens of the phosphate. The crystal may contain a mixture of phosphate and ribose-5-phosphate, which could be modeled into the active site without significant steric clash. Refinement of ribose-5-phosphate alone or at 0.5 occupancy with phosphate as the other 0.5 occupancy resulted in negative density in the |F_o_|-|F_c_| electron density map and high crystallographic *B*-factors for ribose-5-phosphate. Therefore, the final model contains only phosphate. Phosphate is recognized by Ser109, Ser145 and Lys148 of the active site. Some of the negative charge may be stabilized via charge relay from Ser109 to Arg105. In other organisms, Arg105 is a leucine or methionine (Figure 2). The positioning of the phosphate moiety is slightly different than that observed in other structures, such as that of *T. cruzi *RpiB bound to the competitive inhibitor 4-deoxy-4-phospho-D-erythronohydroxamic acid [6] (Figure 4).

Given the substantial differences in the active site of the *C. immitis *RpiB in comparison with other structurally and functionally characterized RpiB enzymes, it is possible that the *C. immitis *enzyme utilizes a substrate different than ribose-5-phosphate/ribulose-5-phosphate. In addition to the single positively charged residue that interacts with the phosphate, the highly conserved histidine involved in ring opening in the catalytic mechanism (His102" in *C. thermocellum *[14]) has been replaced with Ser109" in the *C. immitis *structure (Figure 4). Given the position of the phosphate in the *C. immitis *structure, we suspect that this enzyme may isomerize shorter chain substrates, although confirmation of substrate activity will require further structural and biochemical characterization. Furthermore, *C. immitis *contains another gene (CIMG_09662) that encodes a putative uncharacterized protein which contains 99% sequence identity to RpiA from *C. posadasii *(gene CPC735_023760). Therefore, this essential function of the pentose phosphate pathway may be accomplished by RpiA, allowing RpiB to have evolved with an altered substrate specificity.

### *C. immitis *RpiB covalent inhibition

**Figure 5:**
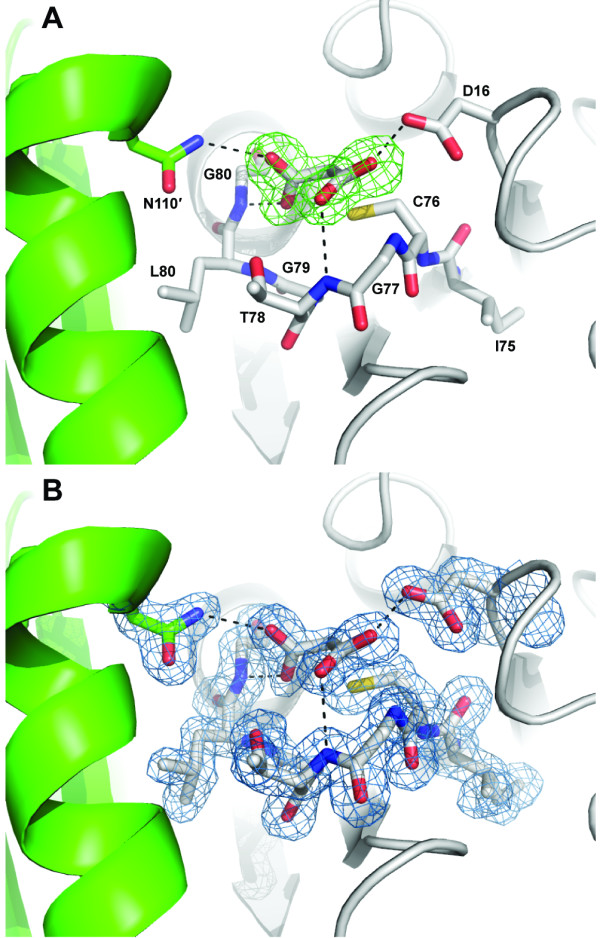
**1.7 Å resolution crystal structure of *C. immitis *RpiB bound to malonic acid**. The coloring is the same as used in earlier figures with one protomer in gray and the other in green. Hydrogen bonds are shown as dashed lines. **A **The |F_o_|-|F_c_| map calculated with model phases lacking the malonic acid residue is shown in green mesh contoured at 3.0 σ. **B **The 2|F_o_|-|F_c_| map calculated with model phases containing the malonic acid residue is shown in blue mesh contoured at 1.0 σ.

The catalytic cysteine residue of RpiB is known to be reactive toward iodoacetate [2,23], although no crystal structure has been determined for an RpiB covalently bound to iodoacetate. Attempts at co-crystallization after incubation with 10 mM iodoacetamide did not yield diffraction quality crystals. However, a 1.7 Å resolution data set was obtained from a crystal grown from MIB buffer (malonic acid, imidazole, boric acid) at pH 5.0 which contained clear evidence for malonic acid tightly bound off Cys76 (Figure 5). The malonic acid refines with a C2-S distance of 2.2 Å, which is longer than that expected for a covalent C-S bond (1.8 Å), but significantly shorter than that expected for van der Waals interactions (>3.3 Å); malonic acid was built into the omit |F_o_|-|F_c_| density and allowed to refine freely in REFMAC5 [24]. Therefore, this structure may reflect the formation of a distorted (long) covalent bond between malonic acid and Cys76. We do not know of a reasonable mechanism for the formation of such a covalent bond between malonic acid and Cys76, which rather seems counterintuitive.

The chemical reaction of Cys76 with iodoacetate is expected to form a Cys-CH_2_COOH adduct. The conformation of either of the carboxylates of malonic acid may be reflective of the carboxylate of the covalent adduct formed with iodoacetate. One of the carboxylates of malonic acid forms hydrogen bonds with the backbone amides of Thr78 (conserved as a serine or threonine, 3.0 Å) and the universally conserved Gly77 (2.9 Å) as well as the side chain of the universally conserved Asp16 (2.6 Å). The hydrogen bond with Asp16 is unlikely to occur at neutral pH where both carboxylates are expected to be deprotonated and negatively charged. In contrast, the interaction is well ordered in the pH 5.0 crystal. Given an approximate pKa of ~4.8, at pH 5.0 about half of the carboxylates should be protonated. The second malonic acid carboxylate forms hydrogen bonding interactions with the backbone amide nitrogen of the universally conserved Gly81 (2.5 Å) and with the side chain of the universally conserved Asn110' of the other protomer of the obligate dimeric enzyme (3.0 Å). The interactions of this carboxylate with RpiB may reflect the interactions formed by the iodoacetate covalent adduct. There is a chloride ion in the active site 3.7 Å away from the malonic acid and near Arg105' and Arg120, although this anion is ill-suited to mimic the iodide ion leaving group of iodoacetate because it is on the opposite side of the caboxylate relative to Cys76. An iodide ion resides in the same place as this chloride ion in both protomers of the asymmetric unit in the iodide-phased structure.

## Conclusions

Here we present crystal structures of RpiB from the pathogenic fungus *C. immitis*, which together with the structures from *T. cruzi *[6] and *G. lamblia *(Edwards, T.E. et al., unpublished) are the only eukaryotic RpiB crystal structures currently available. These structures reveal the presence of a highly reactive cysteine residue in the active site, thought to be the catalytic base in the isomerase reaction and provide insight into a possible structural mechanism for the inhibition of RpiB by iodoacetate. Furthermore, these structures reveal the basis for phosphate recognition by a single positively charged residue and may indicate that this *C. immitis *has altered substrate specificity.

## Methods

### Cloning, expression, and purification

The 163-residue *Coccidioides immitis *putative uncharacterized protein (*C. immitis *RpiB, UniProt ID: P0CL19, formerly Q1DP31; Gene CIMG_07932, targetDB ID: CoimA.00584.a, E. C. 5.3.1.6) was amplified from genomic DNA and cloned into an expression vector (pAVA0421) encoding an N-terminal histidine affinity tag followed by the human rhinovirus 3C protease cleavage sequence using ligation independent cloning [25]. The full length expressed protein contains the tag sequence MAHHHHHHMGTLEAQTQGPGS followed by the 163-residue *C. immitis *RpiB. The plasmid is available through the BEI repository (NR-28451). The plasmid was transformed into *E. coli *BL21 (DE3) R3 Rosetta cells. Starter cultures of LB broth with appropriate antibiotics were grown for ~18 hours at 37°C. Protein was expressed in a LEX bioreactor in ZYP-5052 auto-induction media [26] in the presence of antibiotics in 2 liters of sterilized auto-induction media inoculated with the overnight starter culture. After 24 hours at 25°C the temperature was reduced to 15°C for an additional 60 hours. The sample was centrifuged at 4000 × g for 20 minutes at 4°C. Cell paste was flash frozen in liquid nitrogen and stored at -80°C. The frozen cells were re-suspended in 20 mM HEPES pH 7.0, 300 mM NaCl, 5% w/v glycerol, 0.5% w/v CHAPS, 30 mM imidazole, 10 mM MgCl_2_, 1 mM TCEP, 250 μg/ml AEBSF, 200 μl of lysozyme (100 mg/mL), 5 tablets of Roche protease inhibitor tablets, and 280 μl of β-mercaptoethanol at 4°C. Lysis was achieved by sonication, followed by incubation with Benzonase (20 μL of 25 unit/μL). Insoluble proteins and other cellular components were removed by centrifugation at 10,000 rpm for 60 minutes at 4°C. The supernatant was filtered using a low-protein binding 0.45 μm sterile filter. The soluble fraction was then loaded onto a Ni-NTA His-Trap FF 5 ml column (GE Healthcare). The column was washed with 20 column volumes of wash buffer (20 mM HEPES pH 7.0, 300 mM NaCl, 5% w/v glycerol, 30 mM imidazole, and 1 mM TCEP). The bound protein was eluted with 7 column volumes of elution buffer (20 mM HEPES pH 7.0, 300 mM NaCl, 5% w/v glycerol, 1 mM TCEP, and 500 mM imidazole). The collected protein was loaded onto a Hiload 26/60 Superdex 75 prep grade column (GE Healthcare) equilibrated in SEC buffer (20 mM HEPES pH 7.0, 300 mM NaCl, 5% v/v glycerol, and 1 mM TCEP). The protein eluted off the SEC column in a split peak, which was pooled into two samples and concentrated to 82 mg/mL for the first peak sample and 128 mg/mL for the second peak sample. Both samples were shown by sodium dodecyl sulfate polyacrylamide gel electrophoresis to be >95% pure and contain a protein of the expected molecular weight. Therefore, the two samples may reflect different oligomeric states of the same protein in solution. The sample from the first peak resulted in the crystal structures, whereas the sample from the second peak yielded crystals which did not diffract to better than 3 Å resolution. The purified protein samples were stored at -80°C. Dynamic light scattering (DLS) was performed at 8 mg/mL in SEC buffer on a Malvern Instruments Nano series Zetasizer. Thermofluor experiments were performed in SEC buffer at 2 and 4 mg/mL as described [27].

### Crystallization and structure determination

Crystallization trials were set up according to a crystallization approach [28] using the JCSG+ and PACT sparse matrix screens from Emerald BioSystems. Protein drops (0.4 μL at 82 mg/mL) were diluted with an equal volume of precipitant and equilibrated against 80 μL of precipitant in 96-well sitting drop vapor diffusion format using Compact Junior plates from Emerald BioSystems. A crystal grown from the JCSG+ screen condition B9 (0.1 M Na citrate pH 5.0, 20% w/v PEG 6000) was soaked into a solution containing 0.1 M Na citrate pH 5.0, 20% w/v PEG 6000, 22% v/v ethylene glycol, and 0.7 M NaI for 1 minute, then vitrified in liquid nitrogen. A data set (Table 1) was collected at 100 K under a stream of liquid nitrogen using a Rigaku FR-E+ SuperBright Cu Kα rotating anode X-ray generator with VariMax optics and a Saturn 944+ CCD detector. Data parameters include 360 images, Δφ = 1°, 2θ = 5°, 20 s exposure times, and a detector distance of 50 mm. Data were reduced with XDS [29]. The structure was solved by combined molecular replacement and iodide ion SAD phasing. First, molecular replacement was performed using the protein model from protomer A of *Clostridium thermocellum *crystal structure (PDB ID 3HEE, [14]) as a search model in Phaser [30] from the CCP4 suite [31]. Separately, twenty-one anomalous sites were identified using *phenix.hyss *[32]. Phases calculated from SAD were combined with the MR solution in Phaser EP [30]. After density improvement in parrot [33], the model was initially built using Buccaneer [16] using the default parameters for both programs and Hendrickson Lattman coefficients and R_free _rather than Phi/FOM. Another crystal of *C. immitis *RpiB was grown at 66 mg/mL in the presence of 20 mM ribose-5-phosphate and 12 mM MnCl_2 _in the PACT screen condition A2 (0.1 M SPG buffer pH 5.0, 25% w/v PEG 1500). The crystal was cryo-protected in a solution containing 20 mM ribose-5-phosphate, 12 mM MnCl_2_, 0.1 M SPG buffer pH 4.0, 30% w/v PEG 1500 and 20% v/v ethylene glycol and vitrified. A data set was collected as described above for the iodide crystal with the exception of 2θ = 10°, 340 images and 30 s exposure times. The phosphate bound structure was solved by molecular replacement in Phaser [30] using the protein-only model of the iodide phased structure. A third crystal of *C. immitis *RpiB was grown at 66 mg/mL in the presence of 20 mM ribose-5-phosphate and 12 mM MnCl_2 _in the PACT screen condition B2 (0.1 M MIB buffer pH 5.0, 25% w/v PEG 1500). The crystal was cryo-protected in a solution containing 20 mM ribose-5-phosphate, 12 mM MnCl_2_, 0.1 M MIB buffer pH 4.0, 30% w/v PEG 1500 and 20% v/v ethylene glycol and vitrified. A data set was collected as described above for the iodide crystal with the exception of 2θ = 10°, 360 images and 6 s exposure times. This malonic acid bound structure was solved using the protein model from the phosphate-bound structure. The final models (Table 1) were obtained after numerous rounds of refinement in REFMAC5 [24] and manual re-building in COOT [34]. NCS averaging was not used due to the high resolution of each structure (sub 2 Å). Both 2|F_o_|-|F_c_| and |F_o_|-|F_c_| electron density maps were used in model building. TLS refinement was used with one group per chain. Water molecules were built that were within hydrogen bonding distance to the protein (~3.2 Å) and showed electron density above 1.1 σ in the final 2|F_o_|-|F_c_| electron density map. Structures were assessed for correctness and validated using Molprobity [35]. All diffraction images are freely available (http://www.csgid.org/csgid/pages/diffraction_images).

## Authors' contributions

TEE collected two of the data sets, solved all three structures, analyzed the data and wrote the paper. ABA purified the protein. ERS performed DLS measurements. ROB performed thermofluor analysis. JTL collected one data set. DJL performed large scale expression and KBT performed lysis. MCC verified two structures, and ASG verified the other. BLS is the SSGCID site manager. WCV is an SSGCID Co-PI. PJM is the SSGCID PI. LJS is an SSGCID Co-PI. All authors read and approved the final manuscript.
